# Effect of focused ultrasound neuromodulation of the superior mesenteric plexus on insulin sensitivity and post-operative hyperglycemia in a swine model of surgical stress

**DOI:** 10.1186/s42234-025-00176-7

**Published:** 2025-06-18

**Authors:** Weiguo Song, Khaled Qanud, Dane A. Thompson, Jared M. Huston, Stavros Zanos

**Affiliations:** 1 Institute of Bioelectronic Medicine, Feinstein Institutes for Medical Research at Northwell Health, Manhasset, NY 11030 USA; 2 Department of Surgery, Donald and Barbara Zucker School of Medicine at Hofstra/Northwell, Northwell Health, New Hyde Park, NY 11042 USA; 3 Donald and Barbara Zucker School of Medicine at Hofstra/Northwell, Hempstead, NY 11049 USA; 4 Elmezzi Graduate School of Molecular Medicine at Northwell Health, Manhasset, NY 11030 USA

**Keywords:** Focused ultrasound stimulation, Superior mesenteric plexus, Neuromodulation, Post-operative hyperglycemia

## Abstract

Metabolic stress during major surgery increases insulin resistance and causes post-operative hyperglycemia (POHG), which may in turn contribute to post-operative morbidity and mortality. Intensive insulin therapy for POHG is often ineffective and may even worsen patient outcomes. Non-invasive focused ultrasound stimulation (FUS) of glucose-sensing abdominal neurons improves glucose metabolism in animal models of diabetes, but its potential role in treating POHG remains unknown. In this study, we explored whether FUS of the superior mesenteric plexus (SMP) alters insulin sensitivity and post-operative fasting blood glucose (FBG) in a swine model of surgical stress-induced POHG. In each of 3 anesthetized animals, FUS targeting the porta hepatis (PH) of the liver or the SMP was delivered and insulin sensitivity was assessed in each case. In another series of experiments, 4 animals received SMP-FUS and 3 sham stimulation, after which surgical stress was induced via small bowel resection. In the 7 surgically operated animals, insulin sensitivity was measured before and after SMP-FUS (or sham), and fasting blood glucose (FBG) was measured before and 16 h after surgery. In all animals, insulin sensitivity was assessed using the hyperinsulinemic-euglycemic clamp (HEC) method. Results: SMP-FUS elicits a greater increase in insulin sensitivity than PH-FUS. On the day of surgery, SMP-FUS increases insulin sensitivity, compared to sham treatment. The day after surgery, surgically operated animals develop mild hyperglycemia. SMP-FUS-treated animals have higher FBG than sham-FUS-treated animals. No clear relationship is observed between FUS-induced changes in insulin sensitivity and next-day FBG. Conclusion: While SMP-FUS improves insulin sensitivity during surgery, it may exacerbate POHG.

## Introduction

The stress response to surgery involves immune and metabolic alterations (Desborough 2000), including post-operative hyperglycemia (POHG). POHG arises from stress-induced insulin resistance and results in increased post-operative morbidity and mortality (van den Berghe et al. 2001). Intensive insulin therapy has been employed to manage POHG, but often leads to severe side effects such as hypoglycemia, and may itself increase morbidity and mortality (Investigators et al. 2009). As such, alternative treatment strategies are needed to mitigate these detrimental metabolic responses.

The autonomic nervous system (ANS) modulates glucose metabolism and insulin sensitivity through neural sensing of glucose in the portal circulation and regulation of hepatic glucose production, pancreatic insulin secretion, and peripheral glucose uptake (Ruud et al. 2017). Vagus nerve stimulation (VNS), a widely used autonomic neuromodulation therapy, lowers glucose levels and enhances insulin sensitivity (Joseph et al. 2019). However, VNS therapy requires an implanted device that carries risks of surgical complications (Revesz et al. 2016), as well as undesired effects such as bradycardia and airway symptoms (Chang et al. 2020). Recent advances in non-invasive focused ultrasound (FUS) have created the possibility for modulating neural activity without implantable devices, and potentially offer a safer alternative for targeting inflammation- and glucose-regulating neurons (Cotero et al. 2019, 2022; Ahmed et al. 2022; Ashe et al. 2023; Yaakub et al. 2023; Zanos et al. 2023; Zafeiropoulos et al. 2024).

In this study, we explored whether FUS of glucose-sensing abdominal neurons alters insulin sensitivity and fasting blood glucose in a swine model of POHG.

## Methods and materials

This study utilized a total of 10 adult male Yucatan pigs (approximately 45 kg). 

Initially, 3 healthy animals were used to determine the targeted area for focused ultrasound stimulation (FUS). All animals were placed under general anesthesia. We tested the direct effects of FUS on insulin sensitivity by delivering FUS to either the portal hepatis (PH) of the liver or the superior mesenteric plexus (SMP), for 30 min. When an animal participated in more than one survival experiments, a minimum recovery period of 5 days was observed between procedures. 

Subsequently, we assessed the direct effect of FUS of the SMP on insulin sensitivity and its delayed effect on fasting blood glucose in 7 animals that underwent surgical stress: 4 receiving FUS of the SMP and 3 receiving sham treatment.

All experiments were performed under protocols approved by the Institutional Animal Care and Use Committee of the Feinstein Institutes for Medical Research.

### Hyperinsulinemic-euglycemic clamp (HEC)

**Fig. 1:**
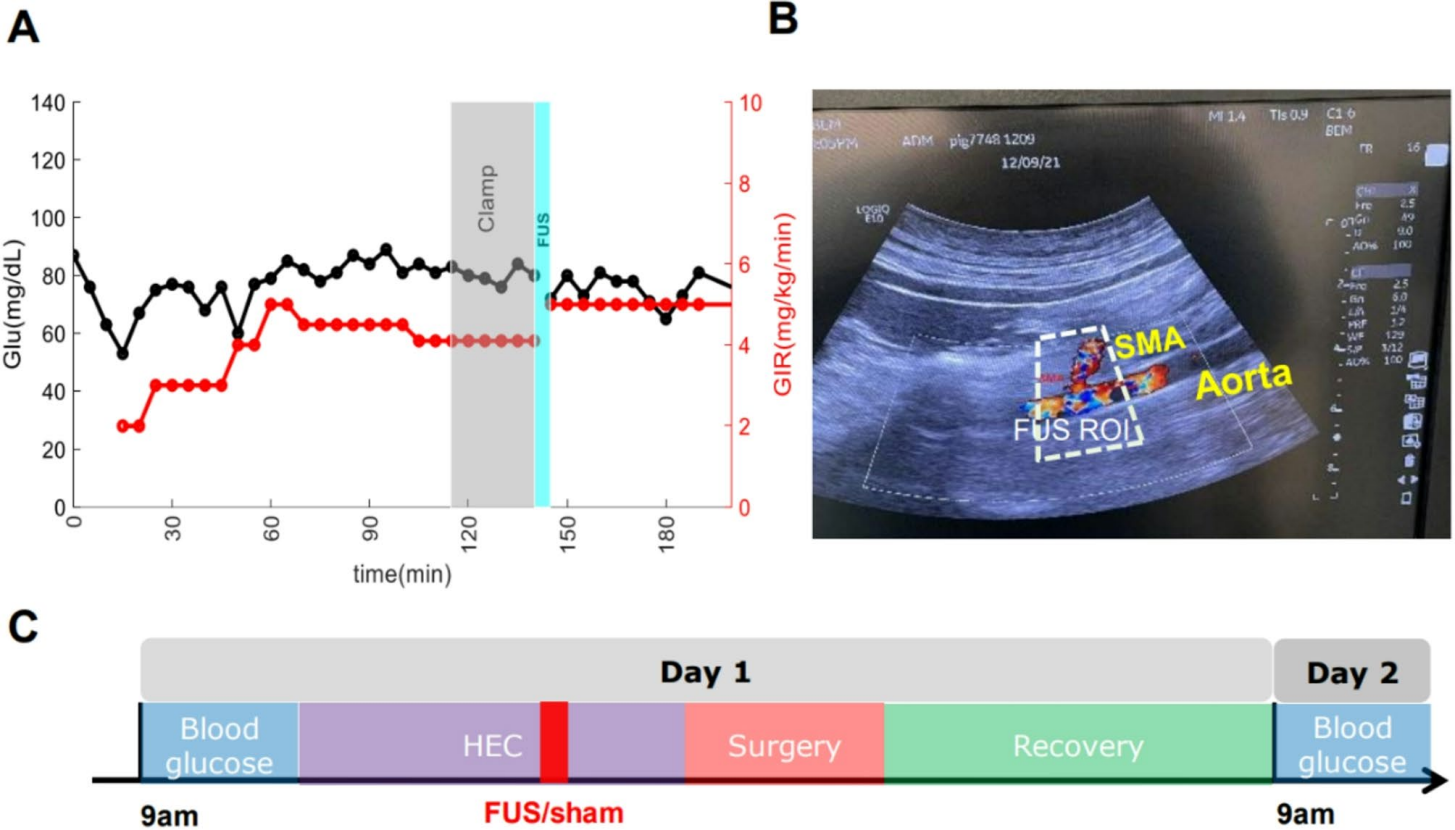
Experimental procedures and timeline. **A**) Example of hyperinsulinemic-euglycemic clamp procedure (HEC). Insulin is infused at a constant rate throughout the procedure, while the glucose infusion rate (GIR) is adjusted to maintain blood glucose levels at a predefined target within 30 min, at which time a “clamp” is established. Afterwards, FUS is delivered to the target site (PH or SMP), and the GIR is readjusted to maintain glucose levels at the clamped level. **(B)** Ultrasound imaging showing that stimulation targeted the SMP located near the superior mesenteric artery (SMA). **(C)** Timeline of the testing protocol in the swine model of surgical stress, in which FUS (or sham) was delivered to the SMP for 30 min, before the surgical procedure was initiated

After placing a central venous line for insulin and glucose infusion and a femoral arterial line for blood sampling, HEC was achieved by adjusting the glucose infusion rate (GIR). The GIR was adjusted every 7 minutes based on blood glucose measurements, while insulin (0.5 mU/kg/min) was continuously infused at a constant rate. A successful clamp was defined as a coefficient of variation in blood glucose levels of less than 10% around a baseline value, for at least 30 min. Following FUS administration, the GIR was adjusted to maintain glucose equilibrium for the next 90 min (Fig. 1A). The direct effect of FUS on insulin sensitivity was determined by assessing changes in GIR from baseline levels.

### Surgically-induced stress

The surgically-induced stress model is associated with post-operative increases in blood glucose (BG) and the development of insulin resistance(Hagve et al. 2015), similar to the response observed in many non-diabetic patients undergoing major surgery (Frisch et al. 2010). With the animal in a supine position, a 15 cm upper midline laparotomy was performed using sharp dissection and monopolar electrocautery. The small bowel was eviscerated and traced distally to the ileocecal valve. Starting at the ileocecal valve, 100 cm of proximal bowel was measured, and the mesentery was opened using electrocautery. The bowel was then divided with a stapler (GIA, 60 mm, 3.8 mm blue load; Covidien). Another 150 cm of proximal bowel was measured and divided again with a stapler. The mesenteric vessels associated with the bowel between the two divisions were ligated prior to excising the bowel. To reconstruct the bowel, the proximal and distal ends of the small bowel were approximated, and small enterotomies were created on the anti-mesenteric sides of each staple line. The two halves of the stapler were inserted through the enterotomies to align the anti-mesenteric sides of the bowel segments, and the stapler was fired to create a side-to-side isoperistaltic small bowel anastomosis. The distal end of the anastomosis was closed with another staple load (TA, 60 mm, 3.5 mm blue load; Covidien). The mesenteric defect was closed with interrupted sutures (4.0 Vicryl), and the small bowel was repositioned within the abdomen. Finally, the fascia was closed in a running fashion (1 PDS), and the skin was closed with staples. The entire surgical procedure typically lasted 2–4 h.

### FUS testing protocols

The ultrasound system (LOGIQ E10, GE Healthcare) enabled us to perform either imaging or FUS using a customized, dual-use, co-registered probe. The maximum output intensity was 3321 W/cm² (pulse width of 200 µs, frequency of 2.27 MHz) at depths of 60 mm within a focal area of 4 × 2.7 mm. Guided by imaging, FUS was delivered to PH or the SMP. First, we tested whether image-guided FUS was site-specific using three healthy animals in multiple survival experiments. To maximize sensitivity in detecting changes in insulin sensitivity, we employed HEC with a low insulin infusion rate (0.5 mU/kg/min). Euglycemia, with a CV of < 10% for at least 30 min, was considered a successful clamp. The animals received FUS to the PH, SMP, or both, and changes in GIR from baseline levels were used to evaluate the effect of FUS on insulin sensitivity. Second, we investigated whether FUS could prevent the development of post-operative hyperglycemia in a surgical stress model. In this study, HEC was established to assess the direct glucose control effect of FUS on SMP, followed by testing its effect in animals subjected to surgical stress. Seven animals were randomized into two groups (SMP-FUS vs. SMP-sham) for a two-day experiment. On the day of surgery (Day 1), blood samples were collected from the femoral artery three times within 30 min, and fasting blood glucose (FBG) was measured. The average of the three measurements was defined as the “glucose level before surgery”. FUS (or sham; placement of probe in a comparable location, without FUS), was applied to the SMP before the abdomen was opened. A standard HEC procedure was followed before, and after FUS (Fig. 1, A). The SMA was visualized using ultrasound imaging (Fig. 1, B), and FUS was applied to the SMP for 30 min. The surgical procedure followed. After the procedure, animals were returned to their cages for the rest of the day and received antibiotics and pain medication. Approximately 16 h after the end of surgery (Day 2), blood samples were collected three times within 30 min from the femoral artery, and FBG was measured. The average of the three measurements was defined as the “glucose level after surgery” (Fig. 1, C). The change in glucose level before and after surgery (day one vs. day two) was assessed using a paired t-test for each group, with a p-value of ≤ 0.05 considered statistically significant.

## Results and discussion

Glucose-sensing neurons have been identified in the portal hepatis (PH) and the superior mesenteric plexus (SMP), with their central axons traveling within the thoracic splanchnic and vagus nerves (Saberi et al. 2008). By activating mechanosensitive receptors on glucose-sensing peripheral neurons, FUS has been shown to suppress hyperglycemia in acute inflammation and in diabetic animal models (Cotero et al. 2019, 2022). While the precise mechanisms behind these actions are still under investigations, FUS may modulate insulin sensitivity via activation of glucose-sensitive afferent pathways and subsequent engagement of neuro-endocrine circuits that regulate glucose homeostasis (Thorens 2024). Studying the effects of FUS neuromodulation on glucose metabolism may provide crucial insights into selective targeting of neural circuits for metabolic modulation and potentially in the treatment of diseases with metabolic dysfunction.

**Fig. 2:**
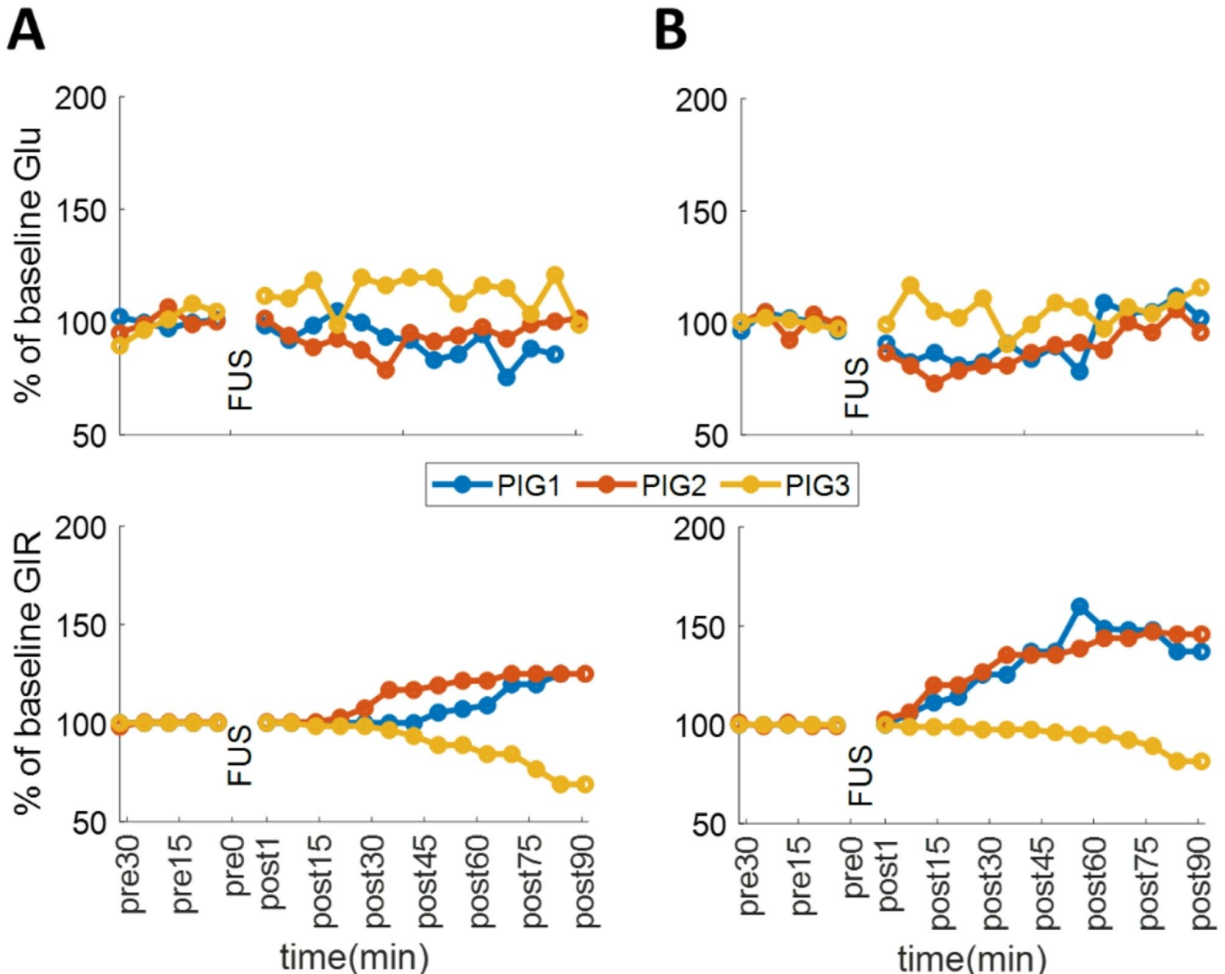
Direct effect of FUS (30 min; 55% maximum intensity), applied either on PH (**A**) or SMP (**B**) on insulin sensitivity assessed through a HEC procedure. Shown separately are traces of % changes of glucose concentrations (top) and traces of GIR (bottom), before, and after FUS in individual animals (*n* = 3)

To evaluate insulin sensitivity independently of the confound of changing glucose levels, we used the hyperinsulinemic, euglycemic clamp (HEC) method. With HEC, blood glucose is maintained at a predefined level by adjusting glucose infusion rate (GIR) against a constant insulin infusion rate (Fig. 1A); the GIR at euglycemic equilibrium assesses insulin sensitivity. We assessed the direct effect of FUS on insulin sensitivity by measuring GIR before, during and after FUS; an increase in GIR with FUS indicates increased insulin sensitivity. In 3 healthy, anesthetized pigs, we found that 30 min of FUS targeting the SMP results in a greater increase in GIR compared to 30 min of FUS targeting the PH (Fig. 2).

PH may regulate blood glucose levels directly, whereas the SMP may influence glucose absorption and regulation indirectly (Fujita and Donovan 2005; Bacharach et al. 2023). The stronger response to FUS targeting the SMP suggests that neurons in the SMP may be more sensitive to FUS than those in the PH or their modulation may lead to more pronounced changes in metabolic homeostasis. This may be explained by different phenotypes of the respective neuronal populations, including expression of different mechanoreceptors, connectivity to central metabolic control centers and connections to other metabolically active organs (Sun et al. 2023). For example, sensory neurons in the PH express TRPA1 + mechanoreceptors and have direct neural connections to the hypothalamus (Cotero et al. 2022), whereas the SMP, through intestinal efferent and afferent projections, may primarily influence intestinal glucose absorption and systemic insulin sensitivity (Fujita and Donovan 2005; Bacharach et al. 2023).

**Fig. 3:**
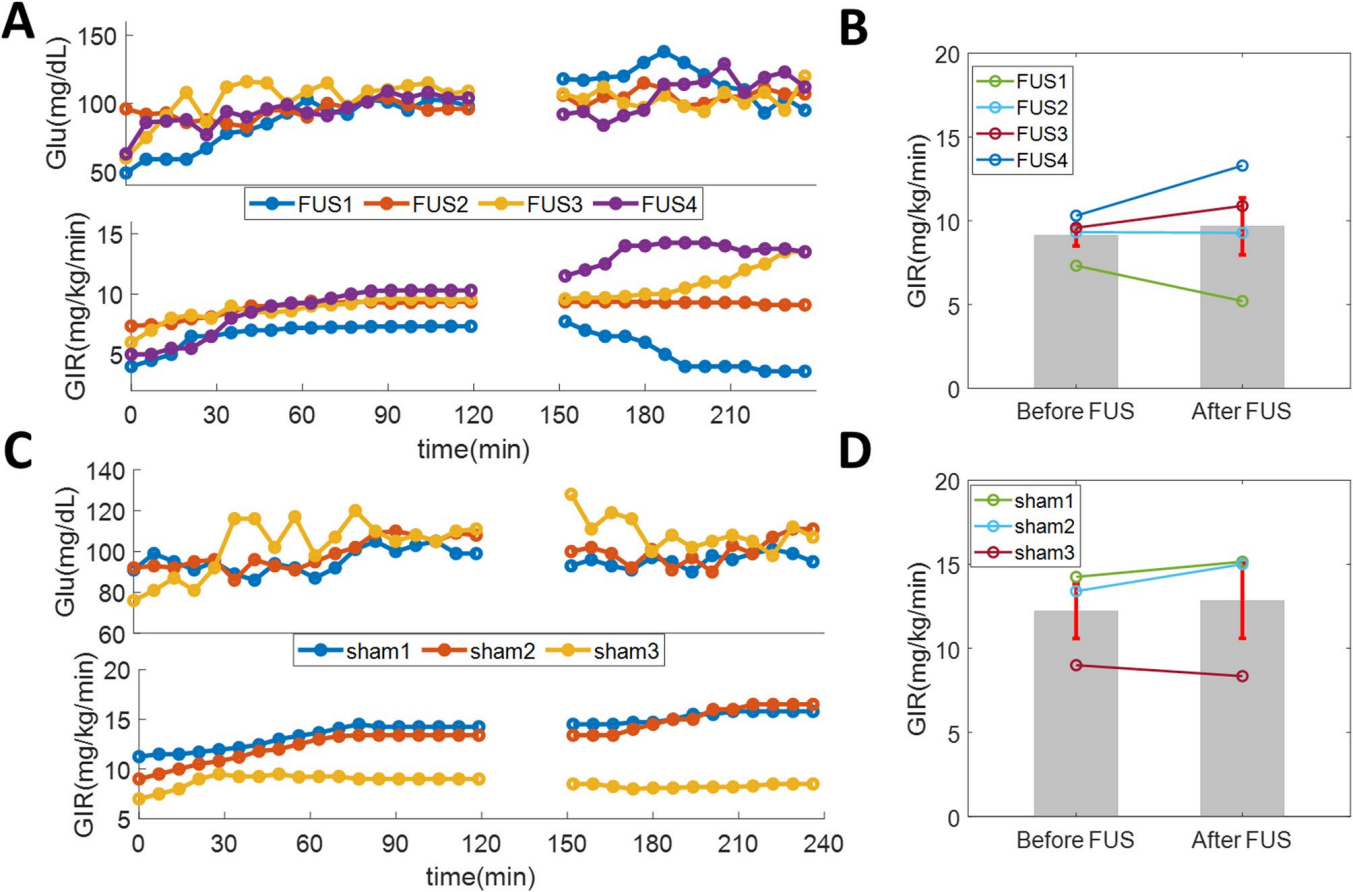
Direct effects of SMP-FUS and sham stimulation on insulin sensitivity, in animals that subsequently underwent surgical stress. (**A**) Blood glucose levels (top panel) and glucose infusion rates (GIR, bottom panel) in 4 animals, before, during and after 30 min of SMP-FUS, under conditions of HEC. Each colored trace represents a different animal. (**B**) GIR before and after FUS, during HEC. (**C**) Same as (**A**), but for animals in which sham stimulation was delivered. (**D**) GIR before and after sham stimulation, during HEC

Based on the results of tests in healthy animals, we selected SMP as the target for FUS in the swine model of surgical stress. To assess the direct effect of SMP-FUS on insulin sensitivity on the day of surgery, HEC equilibrium was established before surgery, followed by 30 min of FUS (or sham stimulation). HEC was maintained during and after FUS, for at least 200 min (Fig. 1C). In the SMP-FUS group, 2 out of 4 animals showed sustained increases in GIR after SMP-FUS, while 1 showed a sustained decrease, and 1 showed no change (Fig. 3A and B). In the sham group, 1 animal showed an increase in GIR, while 2 animals showed no change (Fig. 3C and D).

**Fig. 4:**
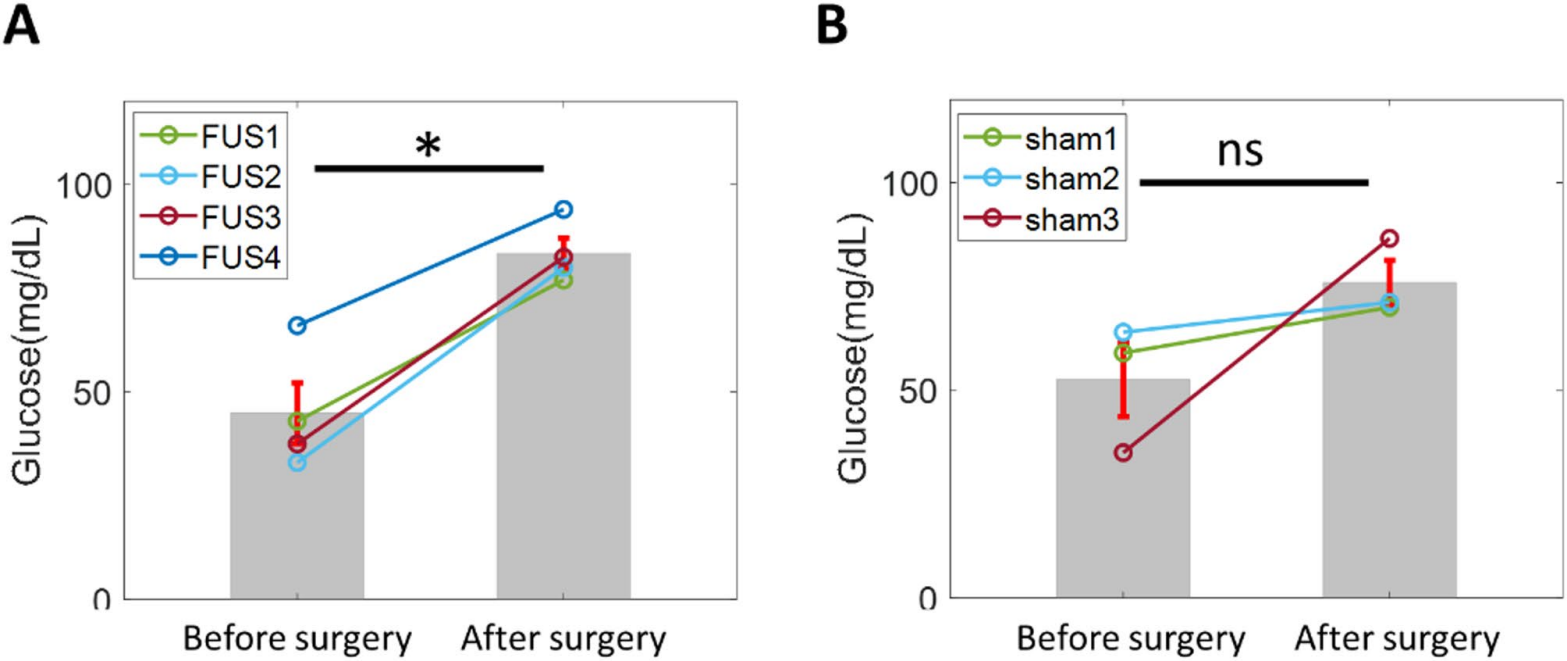
Next-day glycemic response in animals that underwent surgical stress, after either SMP-FUS or SMP-sham stimulation. (**A)** Fasting blood glucose on the day before and on the day after surgery for each animal that received FUS (rank-sum, *p < 0.05). **(B)** Same as (**A**), but for animals that received sham stimulation (rank-sum, p = ns)

After SMP-FUS (or sham), we performed a bowel resection to model surgical stress. To determine the effect of SMP-FUS on post-operative hyperglycemia (POHG), blood samples were collected 16 h after surgery to measure fasting blood glucose (FBG) levels; blood samples for FBG measurement were also collected in the same animals before surgery. In the SMP-FUS-treated group, mean FBG increased from 45 ± 7 mg/dL before surgery to 83 ± 4 mg/dL after surgery (Fig. 4A). In the sham-treated group, the mean glucose level increased from 53 ± 9 mg/dL before surgery to 76 ± 5 mg/dL after surgery (Fig. 4B). Compared with the sham-treated group, the mean increase in fasting blood glucose in the SMP-FUS-treated group was significantly greater (rank-sum test, *p* < 0.05).

In the sham-treated group, there was a trend for negative correlation between GIR during surgery and FBG the following day (Pearson correlation coefficient *r*=-0.97; *p* = 0.15). Despite not being statistically significant (due to small sample size), this finding suggests that animals with higher GIR, and therefore greater insulin sensitivity, preoperatively tend to have lower postoperative FBG levels. In contrast, in the SMP-FUS-treated group, the correlation coefficient was smaller (*r*=-0.23, *p* = 0.77), suggesting that SMP-FUS may have disrupted the relationship between preoperative insulin sensitivity and postoperative FBG. However, due to small sample sizes, these findings need to be replicated in larger studies.

Though preliminary and of small sample size, these results suggest a possible modulation of the complex metabolic responses to surgical stress by SMP-FUS. SMP neurons have been shown to indirectly influence glucose absorption and regulation (Fujita and Donovan 2005; Bacharach et al. 2023) through sympathetic and parasympathetic pathways that modulate digestion, blood flow, and metabolic processes. FUS targeting the SMP may modulate the activity of those autonomic neurons, with a time-dependent effect on fasting blood glucose. SMP-FUS may also affect glucose-sensing signaling from the liver to the brain, as afferent vagal and sympathetic fibers travel through this plexus (Sun MX 2023). The hyperglycemic effect of SMP-FUS in this study of surgical stress contrasts with the hypoglycemic effect of PH-FUS that we have previously documented in models of diabetes (Cotero et al. 2022). Acutely, SMP-FUS produces increase in insulin sensitivity, as assessed by increased GIR after FUS, in most tested animals (Figs. 2 and 3A), similar to PH-FUS (Fig. 2); in that sense SMP-FUS does not appear to be qualitatively different than PH-FUS. It is possible that when FUS is applied in an animal undergoing surgical stress, which constitutes an acute and dynamic metabolic insult, the effects on blood glucose may be more varied and time-dependent than when FUS is applied in a healthy or in a chronically diabetic animal. More frequent measurements of blood glucose, extending over several days after surgery, in FUS- and sham-treated animals, may offer additional insights into the time course of metabolic dysfunction after surgical stress, and the metabolic impact of FUS.

Our study is the first to correlate insulin sensitivity, measured by GIR on the day of surgery, to more sustained metabolic dysfunction, assessed by FBG the day after surgery. The observed trend in correlations suggest that insulin sensitivity before surgical stress may be a predictive marker for POHG. In addition, appropriate pre-operative enhancement of insulin sensitivity could potentially help mitigate perioperative insulin resistance for longer, and reduce POHG. Whether FUS, with additional optimizations, has a role in enhancing insulin sensitivity in this context remains to be seen. In conclusion, given the limited sample size in this study, testing in a larger cohort of animals is required to draw more robust conclusions about the use of neuromodulation in this clinically important problem.

### Limitations

Our small sample size limits our ability to draw definitive conclusions regarding our main findings, and underline the need for more experiments, possibly assessing additional markers biomarkers associated with hyperglycemia (Hill et al. 2024). Additionally, FUS parameters were constrained by inherent hardware limitations of the clinical equipment, which only permits adjustments to output power and treatment duration. Different FUS parameters may exhibit distinct neuromodulation effects, as shown in isolated nerves (Guo et al. 2022) and in behavioral outcomes of transcranial FUS (Murphy et al. 2024). Among these parameters, pulse repetition frequency and pulse duration play significant roles (Fomenko et al. 2020), making them potential targets for further optimization.

## Data Availability

Data is provided within the manuscript.

## Abbreviations

ANS: Autonomic nervous system
BG: Blood glucose
FUS: Focused ultrasound stimulation
GIR: Glucose infusion rate
HEC: Hyperinsulinemic-euglycemic clamp
PH: Porta hepatis
SMP: Superior mesenteric plexus
VNS: Vagus nerve stimulation
